# Methods for detection of viable foodborne pathogens: current state-of-art and future prospects

**DOI:** 10.1007/s00253-020-10542-x

**Published:** 2020-03-26

**Authors:** Antonio C. G. Foddai, Irene R. Grant

**Affiliations:** Institute for Global Food Security, School of Biological Sciences, Queen’s University Belfast, 19 Chlorine Gardens, Belfast, BT9 5DL Northern Ireland, UK

**Keywords:** Cell viability, Detection methods, Foodborne pathogens, Rapid methods, Viable-but-non-culturable

## Abstract

**Abstract:**

The ability to rapidly detect viable pathogens in food is important for public health and food safety reasons. Culture-based detection methods, the traditional means of demonstrating microbial viability, tend to be laborious, time consuming and slow to provide results. Several culture-independent methods to detect viable pathogens have been reported in recent years, including both nucleic acid–based (PCR combined with use of cell viability dyes or reverse-transcriptase PCR to detect messenger RNA) and phage-based (plaque assay or phage amplification and lysis plus PCR/qPCR, immunoassay or enzymatic assay to detect host DNA, progeny phages or intracellular components) methods. Some of these newer methods, particularly phage-based methods, show promise in terms of speed, sensitivity of detection and cost compared with culture for food testing. This review provides an overview of these new approaches and their food testing applications, and discusses their current limitations and future prospects in relation to detection of viable pathogens in food.

**Key points:**

*• Cultural methods may be ‘gold standard’ for assessing viability of pathogens, but they are too slow.*

*• Nucleic acid–based methods offer speed of detection but not consistently proof of cell viability.*

*• Phage-based methods appear to offer best alternative to culture for detecting viable pathogens.*

## Introduction

Many different microorganisms can contaminate foods and cause foodborne illness. Pathogenic bacteria and viruses are responsible for the highest number of foodborne illness outbreaks worldwide (World Health Organisation 2019). Norovirus, hepatitis E virus, *Salmonella* spp., *Campylobacter* spp., *Listeria monocytogenes*, *Staphylococcus aureus* and pathogenic *Escherichia coli* are the main pathogens that cause the highest number of outbreaks linked to food sources (US Centers for Disease Control 2018; Food Standards Agency 2018).

Food business operators need rapid tests to monitor foods for the presence of pathogenic bacteria (Law et al. 2015) or to ensure compliance with legislation stipulating maximum levels of particular pathogens in certain categories of food product (European Commission 2005), to prevent unsafe products reaching the consumer. Border inspection agencies also need rapid tests to detect and prevent the importation of food contaminated with unsafe levels of pathogenic microorganisms, a specified hazard category notified within the Rapid Alert System for Food and Feed (https://ec.europa.eu/food/safety/rasff_en), for example. Tests for foodborne pathogens have historically been culture-based, which is still considered the gold standard (Bhunia 2014). Despite being inexpensive and simple to use, culture-based methods require at least 2–3 days to yield results, and generally must be followed by biochemical tests (‘metabolic fingerprinting’), molecular tests (typically PCR), or mass spectrometry (Ellis et al. 2019), to confirm that the isolate is indeed the pathogen of interest. Due to the perishable nature and, hence, limited shelf-life of many foods, delayed delivery of culture results makes such tests inadequate in many cases. In order to overcome the limitations of culture-based tests, various alternative, and generally more rapid, culture-independent methods to detect viable foodborne pathogens are being proposed. This review will summarise and categorise these methods, focusing exclusively on tests that are capable of demonstrating the viability of foodborne pathogens and highlighting their advantages and limitations. Readers are directed to Bhunia (2014) for a more general review of methods to rapidly detect foodborne pathogens, dead or alive.

## Potential metabolic states of microorganisms in food

Before considering the available methods for detecting viable foodborne pathogens, it is important to understand which metabolic states the term ‘viable’ encompasses. For practicality in food microbiology, viability is commonly understood to mean the ability of bacterial cells to replicate in liquid culture media or to produce a visible colony on solid culture media (Davey 2011). Formation of colonies on a growth medium clearly demonstrates that at least one cell was able to replicate; thus, the cells are alive (Bogosian and Bourneuf 2001; Schottroff et al. 2018). However, the delineation between life and death is a very complex concept, as the route from life to death can include different stages (Davey 2011; Schottroff et al. 2018). Microorganisms in foods exposed to stresses or different environmental conditions can exist in various metabolic states or growth phases and, in some of these states, might transiently lose the ability to grow on or in laboratory culture media. In addition to the fully competent, viable state, the scientific literature suggests that microorganisms in foods, particularly bacteria, may exist in three other metabolic or physiological states—sub-lethally injured, viable-but-non-culturable (VBNC) or ‘persister’, and dormant—the main features of which are summarised in Table 1.

**Table 1:** Main metabolic states that microorganisms may exist in when exposed to food preservation technologies or adverse environmental conditions

Metabolic state	Description and biological features	Induced by	Ability to revert to competent state	References
Injured cells (sub-lethally or severely)	• Damage to essential cell structures and cell functions • Limited ability to grow on selective media; presence solely and predominantly demonstrated on non-selective media	• Prolonged exposure to sub-lethal chemical or physical treatments	Yes/No	Wesche et al. (2009) Li et al. (2014) Espina et.al. (2016)
Viable but non-culturable (VBNC)/‘Persister’	• Low but detectable metabolic activity (genes expressed and proteins produced) • Membrane integrity maintained • Formation of colonies on solid culture media inhibited	• Starvation • Osmotic stress • Oxygen stress • Change in the pH • Exposure to low temperature • Milk pasteurization • Low water activity • Pulsed electric field • Addition of food preservatives • Exposure to disinfectants • Chlorination	Yes	Xu et al. (1982) Kint et al. (2012) Zhao et al. (2017) Kim et al. (2018) Schottroff et al. (2018)
Dormant	• Shutdown of the metabolism • Negligible metabolic activity not detectable by viability assays	• Osmotic stress • Lack of nutrients	Yes	Kell et al. (1998) Setlow (2005)

Many factors, including physical and chemical treatments occurring during food processing, can cause injury to foodborne pathogens (Table 1). Reduced culturability can be the consequence of sub-lethal damage to essential cellular components or lack of essential cellular components, and this damage can be of a temporary or permanent in nature (Kell et al. 1998). Sub-lethally injured bacterial cells in food may not show themselves when plated on selective culture media commonly used for isolation of foodborne pathogens. Given time and conditions appropriate for repair of cellular damage, the cells may be able to recover full viability (Espina et al. 2016). However, if repair of sub-lethal damage is not possible, the cells may enter a VBNC state whilst maintaining their pathogenicity (Wesche et al. 2009).

The VBNC state is generally described as a reversible state, since cells may undergo recovery if suitable conditions occur (Kell et al. 1998; Bogosian and Bourneuf 2001; Ramamurthy et al. 2014). VBNC cells show low but detectable metabolic activity (Mukamolova et al. 2003; Wu et al. 2017). They maintain membrane integrity, express genes and produce proteins (Oliver 2010); however, the formation of colonies on solid culture media is inhibited (Ayrapetyan and Oliver 2016). The VBNC state has been extensively documented in more than 67 pathogenic species including foodborne pathogens such as *Escherichia coli* 0157:H7, *Vibrio* spp., *Listeria monocytogenes*, *Campylobacter jejuni* and *Bacillus cereus* (Zhao et al. 2017), and the existence of VBNC bacteria in food is well documented (Ordax et al. 2009). Increasing scientific evidence suggests some pathogenic microorganisms might enter into a VBNC as an adaptive survival strategy to combat adverse environmental conditions during food processing or preservation. As culture tests rely on the ability of the microorganisms to grow in selective media, VBNC cells might evade detection by culture-based tests, giving rise to false negative results, hence, posing potential risks of human exposure.

Kim et al. (2018) reported that the VBNC state and the ‘persister’ state describe the same dormant phenotype of *Escherichia coli* cells. Kint et al. (2012) defined ‘persister’ cells as the surviving population when a microbial culture is exposed to increasing concentrations of bactericidal antibiotics or to a fixed concentration over a long time. ‘Persister’ cells were formed by *Listeria monocytogenes* cells exposed to nisin (Wu et al. 2017), and potentially, they could occur due to long-term exposure of this pathogen to sanitizers used in the food processing environment. Similar to VBNC cells, ‘persister’ cells show negligible metabolic activity that cannot be detected by viability assays, and they will not probably be culturable. However, upon exposure to specific stimuli, they may regain activity and can thus be cultivated again (Kell et al. 1998). According to Kint et al. (2012), ‘persister’ cells have public health/medical relevance. They probably have no greater implications in terms of food pathogen detection than VBNC cells.

Dormant vegetative cells of foodborne pathogens would be encountered rarely, if ever, in food. However, one of the best-studied types of bacterial dormancy is sporulation (Setlow 2005). Fortunately few of the major pathogens causing foodborne illness have spore-forming ability.

## Methods available to detect viable foodborne pathogens

The methods available to detect viable pathogens in food can be broadly categorised into culture-based and culture-independent (nucleic acid–based and phage-based) methods.

### Culture-based methods

As stated earlier, culture-based methods are generally regarded as the ‘gold standard’ for microbiological analysis of food. Traditional culture relies on the ability of bacteria to grow and multiply on laboratory media and form visible colonies. These methods still represent the first choice for many food testing laboratories as they are sensitive, inexpensive, easy to use, and give either qualitative or quantitative information on the number and type of viable microorganisms present in the food samples (Doyle 2001). However, culture-based analysis of food is generally not a rapid process. A series of steps is required before a definitive identification can be confirmed, which may include pre-enrichment, selective enrichment, plating on selective media, and then biochemical or serological confirmatory tests (Vanderzant and Splittstoesser 1992; Invitski et al. 1999; Bhunia 2014). The entire culture process typically requires 2–3 days for preliminary isolation and up to a week for final confirmation of the species isolated (Zhao et al. 2014). Furthermore, the non-uniform distribution and often low abundance of pathogens in a food sample, the heterogeneity of food matrices, and the presence of indigenous bacteria which might interfere with isolation of specific pathogens can influence the accuracy of culture results (Mandal et al. 2011). Culture-based methods might also have limited detection capability if microorganisms in an injured state or a VBNC state are present in the food being tested.

### Culture-independent methods

There are essentially two culture-independent approaches that represent promising alternatives to culture-based approaches for detection of viable foodborne pathogens, namely nucleic acid–based and bacteriophage-based detection methods. The advantages and limitations of these culture-independent methods are summarised in Table 2.

**Table 2:** Summary of advantages and limitations of culture-independent nucleic acid– and phage-based approaches for detecting or demonstrating the presence of viable pathogens in food

Type of viability test	Method name	Underlying principle	Advantages	Limitations	Example references
Nucleic acid–based	Viability PCR/qPCR	Pre-incubation of test sample with PMA or EMA dyes, which penetrate into bacteria with compromised cell membranes and bind genomic DNA, making it non-amplifiable.	Gives PCR the capability to differentiate viable and dead cells more quickly than culture. qPCR provides quantitative results.	Dead/inactivated bacterial cells do not always have compromised cell membranes, so false positives may result.	Nocker and Camper (2009) Trevors (2012) Emerson et al. (2017)
	Reverse-transcriptase qPCR (RT-qPCR)	Bacterial transcripts are sensitive to degradation by intra- and extra-cellular RNases, so mRNA levels should rapidly decline after cell death. Thus, detectable mRNA would be limited to the viable and active cells within a sample.	Quick compared to culture, but additional cDNA generation step makes it longer test than viability PCR/qPCR.	Not all studies have demonstrated that mRNA is short-lived, so false positive results may occur. RT-qPCR viability assessment validated for longer (> 200 bp) transcription products, but not necessarily short qPCR products.	Techathuvanan et al. (2010) Baskaran et al. (2016) Omori et al. (2017)
Phage-based	Phage amplification (Plaque) assay	Phages only replicate within viable cells and ultimately lyse these cells to release progeny phages within an agar lawn to form plaques (zones of clearing).	A 24-h test, producing countable plaques giving a quantitative result.	Not suited as a high-throughput test. Laborious, multi-step test, which requires cooled molten agar. Virucidal step is key step, otherwise false positive results may be obtained.	Favrin et al. (2003) Botsaris et al. (2010, 2013, 2016) Foddai and Grant (2017) Gerrard et al. (2018)
	Phage amplification + qPCR	As above, but cell lysis occurs in liquid suspension, releasing progeny phages and host DNA, which can both be detected and quantified by qPCR.	Rapid, one-day test, with option to detect released phages or the host DNA by qPCR to demonstrate that lysis has occurred. Only viable cells lyse. Potentially a quantitative assay.	Important that DNA is released into as small a volume as possible to maximize detection sensitivity, otherwise DNA precipitation and column extraction may be necessary.	Sergueev et al. (2010) Anany et al. (2018)
	Phage amplification + immunoassay	Phage amplification proceeds until cell lysis in liquid suspension, releasing progeny phages which can be detected by ELISA or immunochromatographic test	Rapid, one-day test similar to when qPCR is used. Only viable cells lyse. Potentially a quantitative assay.	Analytical sensitivity more limited compared to qPCR detection after phage amplification.	Stewart et al. (2013) Stambach et al. (2015)
	Phage amplification + enzyme assay	Phage amplification proceeds until viable cells burst to release intracellular components such as ATP or ß-galactosidase, which are measured by enzyme assay.	Rapid, one-day test similar to when qPCR or immunoassay are used. Only viable cells lyse. Potentially a quantitative assay.	May require genetically engineered phages. Not many food testing applications to date.	Alcaine et al. (2015a, b) Franche et al. (2017)

### Nucleic acid–based methods

Nucleic acid–based methods operate by detecting specific DNA or RNA sequences of the target pathogenic organism. Polymerase chain reaction, or PCR, is the most commonly used nucleic acid amplification method for detecting pathogenic microorganisms, and over the last two decades, many different advances on the original PCR protocol have been described (Priyanka et al. 2016). However, despite being rapid, specific and sensitive, standard PCR–based detection methods used alone do not provide any indication about the viability of detected cells, as they are not able to discriminate between DNA derived from live as opposed to dead cells. To overcome this limitation, the use of cell viability dyes in combination with DNA amplification methods, sometimes termed viability PCR, has been investigated (Nogva et al. 2003; Rudi et al. 2005; Pan and Breidt 2007). Viability PCR tests are commonly performed using ethidium monoazide (EMA) or propidium monoazide (PMA) dyes. Before any DNA amplification is applied, cells are stained with EMA or PMA, which can only enter perforated cell membranes binding to the DNA. Subsequent exposure of cells to light leads to irreversible damage of nucleic acid resulting in a strong inhibition of PCR amplification. The end result is that only DNA from cells with an intact membrane will be amplified (Nocker and Camper 2009; Trevors 2012; Emerson et al. 2017). Use of viability PCR tests for rapid detection of foodborne pathogens has been extensively explored, and different endpoint detection approaches, but particularly qPCR and Loop-mediated isothermal amplification (LAMP), have been successfully applied (Law et al. 2015; Priyanka et al. 2016). A limitation of the viability PCR approach is that integrity of the cell membrane is not always a reliable indicator of the viability of cells. Evidence suggests that some cells might remain intact even if they do not show any metabolic activity, leading to false positive results (Ayrapetyan and Oliver 2016). Moreover, bacterial cells may have perforated cell walls at some point during their growth, or during cell wall synthesis, so that inhibited DNA amplification in that case might also generate false negative results (Stiefel et al. 2015).

The detection of messenger RNA (mRNA) is considered a better indicator of cell viability than DNA, since this molecule is only present in metabolically active cells (Sheridan et al. 1998). Reverse transcription-PCR (RT-PCR) is one of the RNA-based molecular techniques most commonly used (Lleò et al. 2000). RT-PCR uses the reverse transcriptase enzyme to convert originally extracted mRNA into complementary DNA (cDNA). The newly synthesized cDNA is then used as a template for exponential amplification using conventional PCR (RT-PCR) or quantification using quantitative PCR (RT-qPCR). RT-qPCR appears to be the first choice for the rapid detection of viral foodborne pathogens in food (Morillo et al. 2012; Szabo et al. 2015; Terio et al. 2017). However, it seems that application for detection of bacterial foodborne pathogens is less common and currently appears to be limited to inactivation studies or challenge tests (e.g. Techathuvanan et al. 2010; Baskaran et al. 2016; Omori et al. 2017). This is probably because the method is too laborious, or due to the rapid degradation of RNA in tested samples, which might also lead to false negative results (Xiao et al. 2012).

### Bacteriophage-based methods

The high specificity and natural affinity of bacteriophages, or simply phages, for their host cells make phage-based methods an attractive proposition. Bacteriophages can only replicate inside living cells, meaning that phage-based methods can be tests to demonstrate cell viability (Richter et al. 2018). Schmelcher and Loessner (2014) reviewed the application of bacteriophages for detection of foodborne pathogens more generally.

Most phage-based tests employ lytic phages as lysing agents, and detection of the new progeny phages or intracellular material released from target bacterial cells provides the indication of cell viability. One of the simplest lytic phage-based tests is called the phage amplification assay or simply the plaque assay (Stewart et al. 1998)*.* In this method samples are incubated with seed bacteriophages to start the lytic cycle. Just before the end of the latent period, a chemical virucide (McNerney et al. 1998) or a physical treatment (Oliveira et al. 2012) is applied to kill all the exogenous phages. Just before the burst time, samples are plated with soft agar and an indicator bacterium (either the host bacterium or a fast-growing surrogate host bacterium). Infected bacteria complete the lytic cycle, releasing new phage particles which infect indicator bacteria in their surrounding area, generating zones of clearing or plaques after overnight incubation. The original number of pathogenic bacteria present can be estimated as plaque-forming units (PFU)/ml based on number of plaques formed. Due to the simplicity of the test, and the rapid acquisition of results, the potential use of this approach has been explored for different foodborne pathogens such as *Salmonella* Typhimurium and *Staphylococcus aureus* (Stewart et al. 1998), *Salmonella* Enteritidis and *Escherichia coli* 0157:H7 (Favrin et al. 2001), *Listeria monocytogenes* (Oliveira et al. 2012) and *Mycobacterium avium* subsp. *paratuberculosis* (Foddai et al. 2009). Proof-of-concept for food testing has subsequently been demonstrated for some of these tests (Favrin et al. 2003; Botsaris et al. 2010, 2013, 2016; Foddai and Grant 2017; Gerrard et al. 2018), and promising analytical sensitivity has been observed. However, a key step in the plaque assay is virucide treatment, and if this step is not completely successful, then false positive plaques can be obtained due to survival of some of the seed phages (Stewart et al. 1998; Favrin et al. 2001, 2003; Oliveira et al. 2012). In order to overcome this issue, a further step such as PCR needs to be performed to confirm the identity of DNA at centre of plaques observed (Stanley et al. 2007). This, of course, extends the time to final test result, making these tests more complex and not ideal for many food testing laboratories.

Faster phage-based detection can be achieved by combining the lytic part of the plaque assay and an alternative end-point detection method, such as immunological (Stewart et al. 2013; Stambach et al. 2015) or molecular (Sergueev et al. 2010; Anany et al. 2018) tests to detect either progeny phages or phage DNA, respectively. qPCR appears one of the most promising options. Its use combined to various phage-based lytic methods has demonstrated highly sensitive detection of different pathogens from many different matrices including food (Anany et al. 2018) and clinical samples (Sergueev et al. 2010) within 8 and 4 h, respectively. Use of lateral flow immunochromatography combined to phage lytic methods also demonstrated rapid detection of *Listeria monocytogenes* within 8 h (Stambach et al. 2015). However, the sensitivity of phage immunoassay tests appears slightly lower than phage qPCR tests.

A third type of lytic phage–based method to detect intracellular components released from bacteria also exists. After phage lysis, the quantity of released compounds is monitored through a bioluminescence assay using an enzyme and a substrate. The amount of light generated is proportional to the quantity of intracellular compound released and to the bacterial concentration originally present in samples. Examples of intracellular markers are adenosine-5 triphosphate or ATP (Griffiths 1996; Blasco et al. 1998) and β-galactosidase (Neufeld et al. 2003; Burnham et al. 2014; Chen et al. 2015). More recently, use of engineered lysogenic bacteriophages has also permitted the development of other prototype phage–based tests using a wider range of enzymatic reactions, including luciferase-based (Zhang et al. 2016; Franche et al. 2017), protease-based (Alcaine et al. 2015a), and alkaline phosphatase–based (Alcaine et al. 2015b) phage detection methods. Finally, as released intracellular content is highly conductive, changes of conductivity in the surrounding environment can be used as a signal of viable bacteria present in the sample. Conductivity variation generated by lysed bacteria can be detected using impedance spectroscopy combined with specifically designed microfluidic chambers (Mortari et al. 2015). Some of these phage-based assays have already demonstrated rapid and sensitive detection from broth culture (Neufeld et al. 2003; Mortari et al. 2015), water (Burnham et al. 2014) or drinking water (Chen et al. 2015). Currently, little information seems to be available regarding their application for food testing.

## Conclusions

More rapid and sensitive tests for detection of viable pathogens in food are continually being sought. Culture-based methods are becoming too laborious and time consuming to apply, and might have limited detection capability if pathogens in a VBNC state are present in food. Molecular tests, particularly mRNA-based tests, represent a potential solution for the rapid detection of living microorganisms. However, the perishable nature of mRNA still represents a barrier to the large-scale use of reverse transcriptase PCR for food testing purposes. A range of lytic phage–based methodologies have emerged over the last two decades, which are exhibiting high detection sensitivity for several foodborne pathogens in many different matrices including food and water. The combination of phage amplification and lysis with PCR/qPCR, immunoassay or enzyme assay endpoint detection approaches seems to be the most promising rapid alternative to cultural methods for detection of viable pathogens in food. Providing host cell metabolism is occurring, phage amplification will take place and pathogen cells will eventually burst to release measurable intracellular components such as ATP, enzymes, host DNA or progeny phages.
